# High expression of long non-coding RNA SBF2-AS1 promotes proliferation in non-small cell lung cancer

**DOI:** 10.1186/s13046-016-0352-9

**Published:** 2016-05-06

**Authors:** Junjie Lv, Mantang Qiu, Wenjia Xia, Chao Liu, Youtao Xu, Jie Wang, Xuechun Leng, Su Huang, Rong Zhu, Ming Zhao, Fengqing Ji, Lin Xu, Keping Xu, Rong Yin

**Affiliations:** Department of Thoracic Surgery, Huai’an First People’s Hospital, Nanjing Medical University, 6 West Beijing Rd, Huai’an, 223300 China; Department of Thoracic Surgery, Nanjing Medical University Affiliated Cancer Hospital, Jiangsu Key Laboratory of Molecular and Translational Cancer Research, Cancer Institute of Jiangsu Province, Nanjing, 210009 China; The Fourth Clinical College of Nanjing Medical University, Nanjing, 210000 China

**Keywords:** lncRNA, SBF2-AS1, NSCLC, Proliferation, Epigenetic regulation

## Abstract

**Background:**

Recent evidence has proven that long noncoding RNAs (lncRNAs) play important roles in cancer biology, while few lncRNAs have been characterized in NSCLC. Here, we characterized a novel lncRNA, SBF2 antisense RNA 1 (SBF2-AS1), in non-small cell lung cancer (NSCLC).

**Methods:**

Quantitative real-time PCR was used to quantify SBF2-AS1 expression in NSCLC tissues and cell lines. The correlation of SBF2-AS1 expression with clinicopathologic features was analyzed in a cohort NSCLC patient. Loss of function and gain of function studies were performed to determine the effects of SBF2-AS1 on proliferation and metastasis of NSCLC cells. RNA immunoprecipitation and chromosome immunoprecipitation assay was performed to confirm the interaction between SBF2-AS1 with protein and chromosome.

**Results:**

We confirmed that SBF2-AS1 was significantly upregulated in NSCLC compared with corresponding non-tumor tissues, and a high expression level of SBF2-AS1 was correlated with lymph node metastasis and advanced TNM stage. Using siRNAs specifically targeting SBF2-AS1 and plasmid vector, we successfully silenced and overexpressed SBF2-AS1 in NSCCLC cell lines and investigated its biological function both in vitro and in vivo. After the silencing of SBF2-AS1, the metastasis of NSCLC cells was significantly inhibited, the silencing of SBF2-AS1 decreased the proliferation of NSCLC cells, and the cell cycle was arrested at the G1 phase; while overexpression promoted proliferation ability. Xenograft tumor models revealed that the silencing of SBF2-AS1 inhibited tumor growth in vivo. We speculated that SBF2-AS1 might negatively regulate P21. RNA immunoprecipitation discovered that SBF2-AS2 could bind with a core component of polycomb repressive complex2, SUZ12. Additionally chromatin immunoprecipitation assay demonstrated that, after silencing SBF2-AS1, the enrichment of SUZ12 and trimethylation of histone 3 lysine 27 decreased at the promoter region of P21.

**Conclusions:**

We demonstrated that SBF2-AS1 is upregulated in NSCLC and promotes proliferation of NSCLC tumor cells. SBF2-AS1 may serve as a novel biomarker and potential therapeutic target for NSCLC patients.

## Background

In the past decades, lung cancer has been the leading cause of cancer-related death worldwide [1]. Non-small cell lung cancer (NSCLC) is the most common type of lung cancer, which consists of two most common histological types, squamous cell carcinoma and adenocarcinoma. To date, the improvements in the treatment of lung cancer have been achieved by the development of combined treatments, such as surgical resection, systemic chemotherapy and targeted drugs. However, the overall five-year survival rate of NSCLC remains unsatisfactory [2]. Therefore, to develop more effective treatment methods, it is urgent to fully discover the genetic and molecular features of NSCLC.

Recently, evidence has shown that at least 90 % of the total mammalian genome is actively transcribed [3]. However, only approximately 1.5 % of the genome sequence comprise protein-coding genes [4]. Non-protein-coding RNA (ncRNA) transcripts consist of >98 % of the mammalian transcriptome and were once thought to be “junk” or “transcription noise”. Recent evidence has proven that ncRNAs—for example microRNAs—play significant roles in various biological processes [4–6]. Long noncoding RNAs (lncRNAs), ncRNAs larger than 200 nucleotides, play a crucial role in diverse cellular processes such as cell growth [7], differentiation [8], the immune response [9], and cancer metastasis [10–12].

By analyzing a published lncRNA microarray dataset of NSCLC [13], we found that the novel lncRNA SBF2 antisense RNA 1 (SBF2-AS1) was significantly upregulated in NSCLC tissues compared with the corresponding non-tumor tissues. SBF2-AS1 is a 2708 nt antisense RNA to SBF2, which is located at the 11p15.1 locus. However, the expression profile and potential function of SBF2-AS1 in NSCLC remain unknown. In the present study, we validated the upregulation of SBF2-AS1 in NSCLC and found that a high expression level of SBF2-AS1 was correlated with advanced TNM stage. Using small interfering RNA (siRNA)-mediated silencing of SBF2-AS1, NSCLC cell proliferation was inhibited both in vivo and in vitro.

## Methods

### Patients and tissue samples

Primary NSCLC tissues and adjoining normal tissues were collected from patients received surgical resection of NSCLC from 2012 to 2014 at the Department of Thoracic Surgery, Cancer Institute of Jiangsu Province. All patients did not receive radiotherapy or chemotherapy before surgical resection. All tumor specimens and adjoining normal specimens were snap-frozen immediately after resection, and stored in liquid nitrogen until total RNA extraction. All tumor and paired normal tissues were verified by experienced pathologists. Clinical characteristics were also collected for each patient, and informed written consents were obtained from all patients included in this research. This study was approved by the Ethics Boards of the Cancer Institute of Jiangsu Province.

### Cell lines and culture conditions

All cell lines (A549, NCI-H1975, NCI-H358, NCI-H1299, SPC-A1, and human bronchial epithelial cell (HBE)) were purchased from Shanghai Institutes for Biological Science, China. NCI-H1975, A549 and NCI-H1299 cells were cultured in RPMI 1640 medium (KeyGene, Nanjing, China), NCI-H358, SPC-A1, and HBE cells were cultured in DMEM medium (KeyGene, Nanjing, China), supplemented with 10 % fetal bovine serum with 100U/ml penicillin and 100 mg/ml streptomycin included. All cell lines were grown in humidified air at 37 °C with 5 % CO_2_.

### RNA extraction and qRT-PCR analyses

Total RNA was isolated with TRIzol reagent (Life Technologies, Scotland, UK) according to the manufacturer’s protocol. About 1.0ug total RNA was reverse transcribed in a final volume of 20ul using the PrimeScript RT Master Mix (Takara, Cat.#RR036a) according to the manufacturer’s protocol. After reverse transcription, the quantitative real-time polymerase chain reaction (qRT-PCR) was carried out using the SYBR Select Master Mix (Applied Biosystems, cat: 4472908) with 0.5ul cDNA on ABI 7900 system (Applied Biosystems, Foster City, CA, USA) according to the manufacturer’s instructions. The relative levels of SBF2-AS1 were confirmed by qRT-PCR. Glyceraldehyde-3-phosphate dehydrogenase (GAPDH) and β-actin were measured as internal controls. The qRT-PCR reaction was implemented under the following conditions: 95 °C for 10 min, 40 cycles of 95 °C for 15 s, and 60 °C for 1 min. The fold changes of individual genes were calculated by 2-ΔΔCt methods [14]. QRT-PCR primers used were: 5′-GGACTAGTGGAGAAGGTGCG-3′ (Forward) and 5′-GGGCGCTGCCCATCATCATG-3′ (Reverse) for P15, 5′-AAACTTGGAAATCCCGAGATTGC-3′ (Forward) and 5′-CGAAACCAGTTCGGTCTTTCAA-3′ (Reverse) for P18, 5′-AGACCATGTGGACCTGTCACTG-3′ (Forward) and 5′-GTTTGGAGTGGTAGAAATCTGTC-3′ (Reverse) for P21, 5′-TGCAACCGACGATTCTTCTACTCAA-3′ (Forward) and 5′-CAAGCAGTGATGTATCTGATAAACAAGG-3′ (Reverse) for P27, 5′-CACCGAATAGTTACGGTCGG-3′ (Forward) and 5′-GCACGGGTCGGGTGAGAGTG-3′ (Reverse) for P16, 5′-AGGCTGACCACGAGCTTTTC-3′ (Forward) and 5′-GGTGCTATGAGATTCCGAGTTC-3′ (Reverse) for SUZ12, 5′-GAAATCGTGCGTGACATTAA-3′ (Forward) and 5′-AAGGAAGGCTGGAAGAGTG-3′ (Reverse) for β-actin, and 5′-CCACATCGCTCAGACACCAT -3′ (Forward) and 5′-ACCAGGCGCCCAATACG -3′ (Reverse) for GAPDH.

### Western blot assay

Cells were harvested, and protein was extracted from transfected cells and quantified as previously described49 using 12 % or 4 ~ 20 % polyacrylamide gradient SDS gel. Anti-β-actin and anti-SUZ12 were from Abcam (Hong Kong, China). Anti-P21 and anti-Cyclin D1 were from Cell Signaling Technology (Boston, MA, USA).

### siRNA and plasmid transfection of NSCLC cells

A549 and H1975 cells were planted in six-well plate 24 h before transfection. When they were about 70 % confluent, cells were transfected with siRNA targeting specific genes or negative control (RealGene, Nanjing, China) by using the Lipofectamine RNAimax reagent (Invitrogen, USA) according to the protocol provided by the manufacturer. The siRNA sequences for SBF2-AS1 were 5′-CAGAAGGAGUCUACUGCUAAG-3′ (Sense) and 5′-UAGCAGUAGACUCCUUCUGGG-3′ (Antisense) for 204 site and 5′-GCAAGCCUGCAUGGUACAUTT -3′ (Sense) and 5′-AUGUACCAUGCAGGCUUGCTT -3′ (Antisense) for 1021 site. The siRNA sequences for SUZ12 were 5′-GUCGCAACGGACCAGUUAATT-3′ (Sense) and 5′-UUAACUGGUCCGUUGCGACTT-3′ (Antisense).

The SBF2-AS1 sequence was synthesized according to the full-length SBF2-AS1 sequence (based on the GAS5 sequence, NR_002578, in NCBI) and then subcloned into a pCDNA3.1 vector (Invitrogen, Shanghai, China). The pCDNA-GAS5 or empty vector was transfected into SPC-A1 cells using Lipofectamine 3000 reagent (Invitrogen, USA), according to the manufacturer’s instructions. The empty pcDNA3.1 vector was used as the control.

### Cell proliferation assay

Cell proliferation was assayed by Cell Counting Kit-8 (CCK8) assay (Promega). The transfected cells were plated in 96-well plates (4000cells per well) 24 h after transfection and cultured at 37 °C and 5 % CO_2_ atmosphere. CCK8 assay was used to detect the relative cell growth every 24 h according to the instructions of manufacturer. Simply, 20ul of CCK8 solution was added to each well, and each well was measured spectrophotometrically at 450 nm after incubating for 2 h.

### Cell migration and invasion assays

For migration assay, transfected cells (3 * 10^5^) were plated in the upper chamber of transwell assay inserts (8 mm pores, Millipore, Billerica, MA) containing 200ul of serum-free 1640 medium. The lower chambers were filled with 1640 containing 10 % FBS. After 24 h of incubation, the cells on the filter surface were fixed with methanol, stained with crystal violet, and photographed. Migration was assessed by counting the number of stained cell nuclei from 5 random fields per filter in each group.

For invasion assay, transfected cells (5 * 10^5^) were plated in the top chamber with a matrigel-coated membrane (BD Biosciences) in 500ul serum-free 1640. Also, the bottom chambers were filled with conditioned 1640. The invasion function was determined after incubating 48 h as mentioned previously in migration.

### Flow-cytometric analysis

Transfected cells were harvested after transfection by trypsinization. After the double staining with fluorescein isothiocyanate (FITC)-Annexin V and propidium iodide was done by the FITC Annexin V Apoptosis Detection Kit (BD Biosciences) according to the manufacturer’s recommendations. The cells were analyzed with a flow cytometry (FACScan; BD Biosciences) equipped with a Cell Quest software (BD Biosciences). Cells for cell-cycle analysis were stained with propidium oxide by the Cycle TEST PLUS DNA Reagent Kit (BD Biosciences) following the protocol and analyzed by FACScan. The percentage of the cells in G1, S, and G2–M phase were counted and compared.

### RNA immunoprecipitation (RIP)

RIP experiments were performed using a Magna RIP RNA-Binding Protein Immunoprecipitation Kit (Millipore) according to the manufacturer’s instructions. Antibodies of EZH2 and SUZ12 were from Abcam.

### Chromatin immunoprecipitation (ChIP) assays

The ChIP assays were performed using EZ-CHIP KIT according to the manufacturer’s instruction (Millipore, Billerica, MA, USA). H3K27 antibody was from Millipore. The ChIP primers for the promoter region of P21 were as follows: 5′-GCCTTCCTCACATCCTCC-3′ (Forward) and 5′-CAAGAGTGCCCAGTCCAG-3′ (Reverse).

### Xenograft experiment

Transient transfection was performed in A549 cells with shLUADT1 or scrambled control sequence using Lipofectamine 2000 (Invitrogen). After 48 h of transfection, the cells were collected and injected into either side of the posterior flank of the same male BALB/c nude mouse. The tumor volumes and weights were measured every 2 days in the mice; the tumor volumes were measured as length × width^2^ × 0.5. Sixteen days after injection, the mice were sacrificed, the tumor weights were measured, and the tumors were collected for further analysis. The LUADT1 levels were determined by qRT-PCR.

### Immunohistochemistry

Xenograft tumor tissue samples were immunostained for p27 and Ki67. Anti-Ki67 was from Santa Cruz Biotechnology.

### Statistical analysis

Student’s *t*-test, one-way ANOVA analysis, and Spearman test were performed to analyze the data using SPSS 18.0 software. *P* < 0.05 was considered statistically significant.

## Results

### Upregulation of SBF2-AS1 in NSCLC

**Fig 1:**
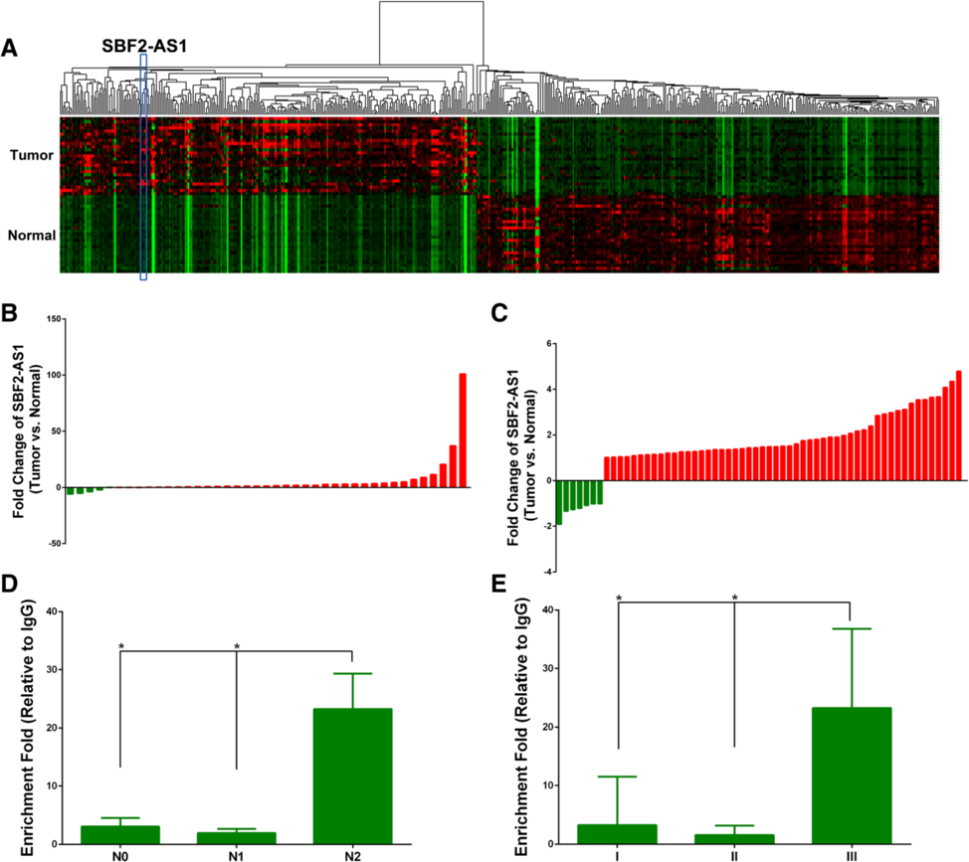
SBF2-AS1 is overexpressed in NSCLC tissues. Expression heatmap of differentially expressed genes in NSCLC (**a**) and SBF2-AS1 was highlighted. Expression level of SBF2-AS1 in a cohort of NSCLC patients (**b**) and the GSE19804 dataset (**c**). Red: upregulation, green: downregulation. Differential expression level of SBF2-AS1 between the different lymph nodes (**d**) and TNM (**e**) stages. * *P* < 0.05

Inspired by a previous study that analyzed the lncRNA expression profile of NSCLC in Chinese patients [13], we found that the novel lncRNA SBF2-AS1 was upregulated in lung adenocarcinoma (Fig. 1a). In an expression cohort of 41 lung cancer patients, we confirmed that SBF2-AS1 was significantly upregulated in lung cancer patients compared with paired adjacent non-tumor tissues (*P* < 0.01). As shown in Fig. 1b, upregulation of SBF2-AS1 was observed in 36 of 41 patients with an average fold increase of 5.36. In addition to our expression cohort, we also validated the overexpression of SBF2-AS1 in another set of microarrays of lung cancer patients from the Gene Expression Omnibus (GSE19804) [15]. As shown in Fig. 1c, the expression of SBF2-AS1 was quite homogeneous, and overexpression was found in 53 of 60 cases.

We further analyzed the relationships between the SBF2-AS1 expression levels and clinicopathological features of NSCLC patients. As shown in Table 1, there was no significant difference between gender, age, or smoking status and the expression level of SBF2-AS1. However, the expression level of SBF2-AS1 was significantly correlated with lymph node stage (*P* = 0.032, Fig. 1d) and TNM stage (*P* = 0.047, Fig. 1e), and a higher expressed level indicated advanced stage. Thus, these lines of evidence demonstrated that SBF2-AS1 was upregulated in NSCLC and correlated with advanced TNM stage.

**Table 1:** Relationship between SBF2-AS1 expression and clinicopathological characteristics

Groups		Upregulation Fold	Number of Patients	*P* Value
Gender	Male	7.57	19	0.442
	Female	3.46	22	
Age	≤65	5.90	32	0.55
	>65	3.45	9	
Smoke	No	5.96	34	0.62
	Yes	2.45	7	
N Stage	0	3.06	30	0.032^a^
	1	1.96	6	
	2	23.27	5	
T Stage	1	3.60	28	0.436
	2	9.89	12	
	3	0.41	1	
TNM Stage	I	3.25	28	0.047^a^
	II	1.57	8	
	III	23.27	5	

^a^Significant association

### Silencing of SBF2-AS1 inhibited the proliferation and metastasis of NSCLC cells

**Fig 2:**
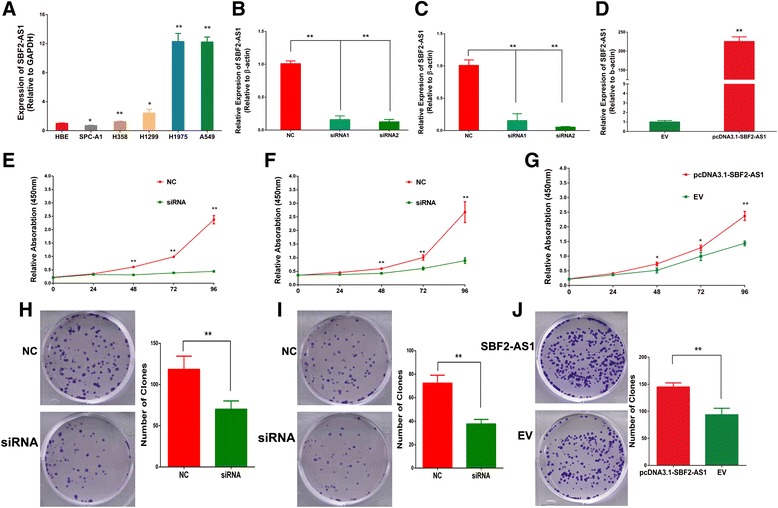
SBF2-AS1 is overexpressed in most NSCLC cell lines compared with the normal human bronchus epithelium cell line HBE (**a**). SBF2-AS1 was efficiently inhibited by siRNA in the A549 (**b**) and H1975 (**c**) cell lines compared with NC and pcDNA3.1-SBF2-AS1 successfully overexpressed SBF2-AS1 in SPC-A1 cells (**d**) compared with empty vector (EV). After the silencing of SBF2-AS1, proliferation and colony formation ability were significantly inhibited in the A549 (**e**, **h**) and H1975 (**f**, **i**) cell lines, while overexpression of SBF2-AS1 increased proliferation ability and colony formation ability in SPC-A1 cells (**g**, **j**). * *P* < 0.05, **P < 0.01

We compared the expression of SBF2-AS1 between NSCLC cell lines and human bronchial epithelial (HBE) cells, and the results revealed that SBF2-AS1 was upregulated in most NSCLC cell lines and showed the highest expression level in A549 and H1975 cells, while the expression in SPC-A1 cell line was lower (Fig. 2a). Thus, A549,H1975, and SPC-A1 were utilized as cell models to investigate the biological function of SBF2-AS1. Two small interfering RNA sequences were designed to specifically target SBF2-AS1 at the 204 (siRNA1) and 1021 (siRNA2) sites. As shown, siRNA2 showed better inhibition efficacy, and siRNA2 was used in subsequent experiments (Fig. 2b, c). Additionally, expression of SBF2-AS1 was significantly over-expressed in SPC-A1 cells by plasmid (Fig. 2d).

**Fig 3:**
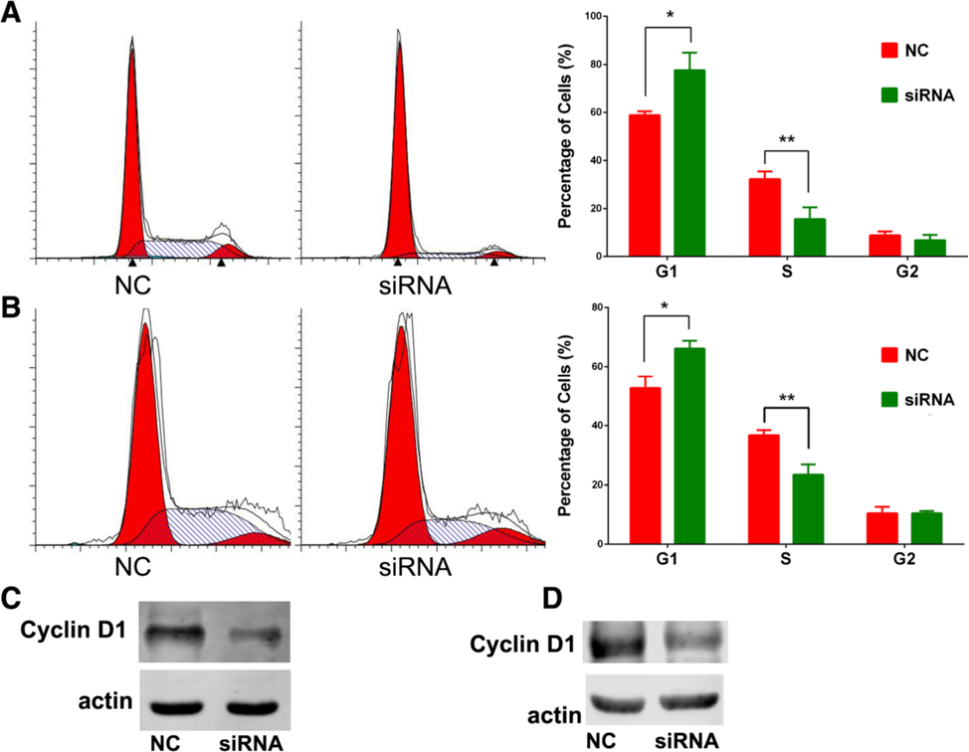
Cell cycle analysis was performed after the silencing of SBF2-AS1 in the A549 (**a**) and H1975 (**b**) cell lines. Significant G1 phase arrest and S phase decrease were observed in both cell lines. Western blotting showed that the Cyclin D1 protein level was decreased after the silencing of SBF2-AS1 in A549 cells (**c**) and H1975 cells (**d**)

**Fig. 4:**
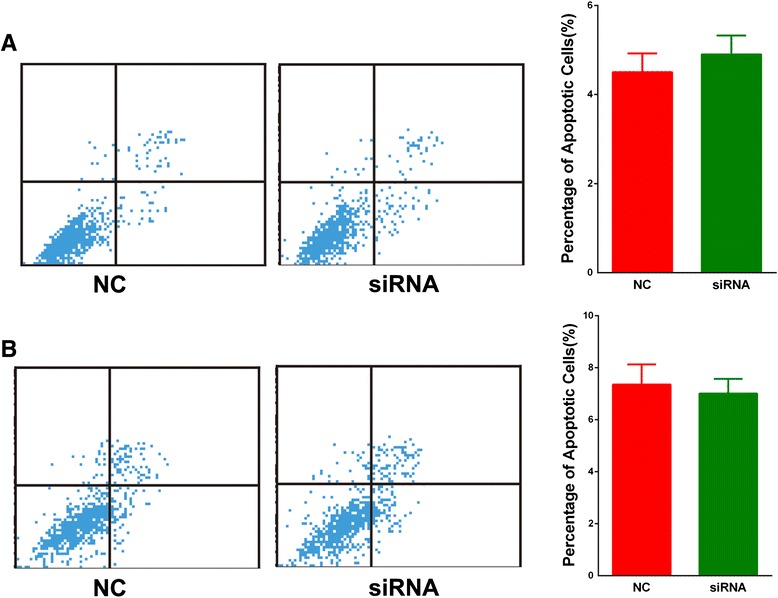
Apoptosis is not affected by the silencing of SBF2-AS1 in the A549 (**a**) or H1975 (**b**) cell lines

Cell proliferation ability was first analyzed. The CCK8 assay showed that, after silencing of SBF2-AS1, the proliferation ability of A549 and H1975 cells was significantly decreased, and the inhibition was more remarkable in A549 cells, while overexpression of SBF2-AS1 increased proliferation ability of SPC-A1 cells (Fig. 2e, f, g). Next, colony formation assay was conducted. Compared with the negative control, siRNA treatment significantly inhibited the colony formation ability of NSCLC cell lines (Fig. 2h, i). In contrast, overexpression of SBF2-AS1 increased the number of colonies in SPC-A1 cells compared with empty vector. After treatment with NC and siRNA, the cells were stained with propidium iodide and analyzed by flow cytometry. We observed significant cell cycle arrest at the G1 phase with a remarkable decrease in S phase (Fig. 3a, b). To support the flow cytometry results (Fig. 3c), Western blotting was performed and revealed that Cyclin D1 was also decreased after the silencing of SBF2-AS1. Additionally, apoptosis was investigated by flow cytometry; no statistically significant difference was found (Fig. 4). Therefore, these results showed that the silencing of SBF2-AS1 inhibited the proliferation ability of NSCLC cells through cell cycle arrest.

**Fig. 5:**
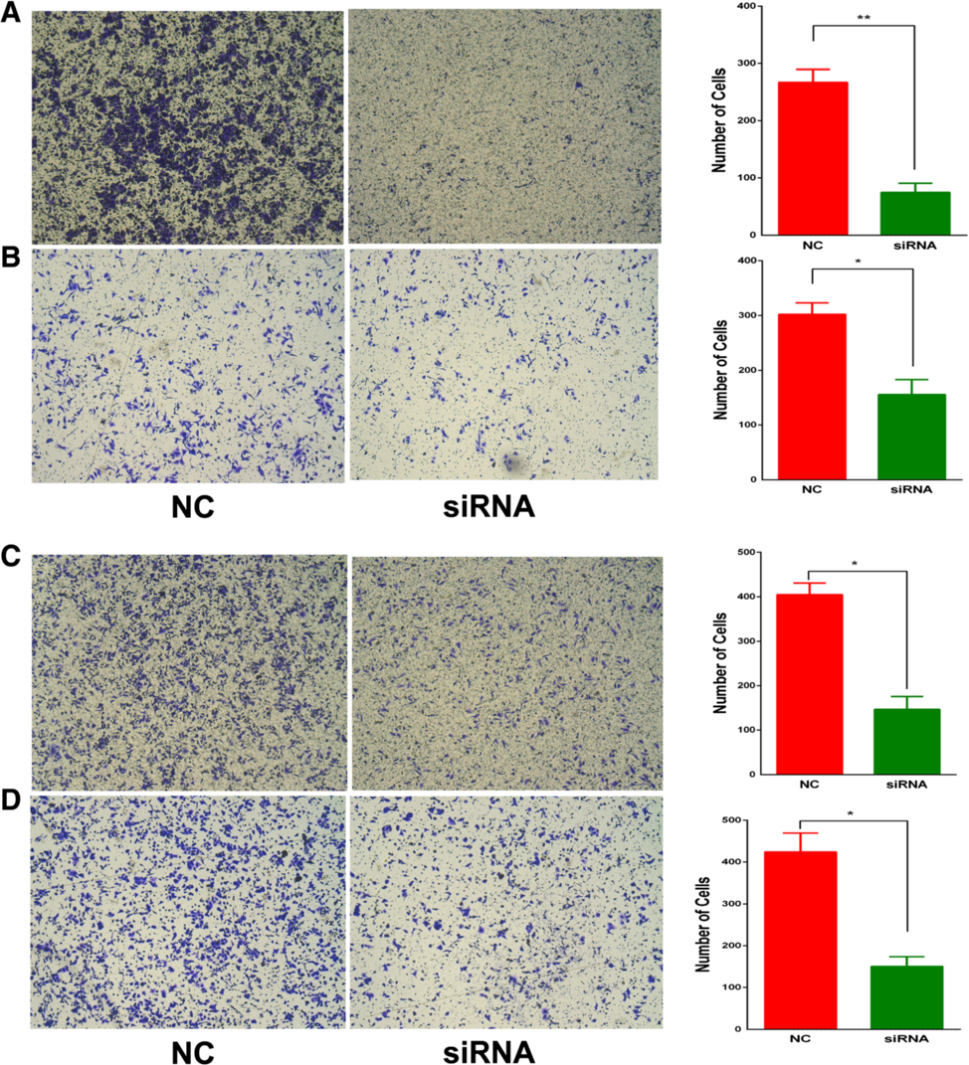
Transwell and migration assays showed that metastasis was inhibited by the silencing of SBF2-AS1 in the A549 (**a**, **c**) and H1975 (**b**, **d**) cell lines

We further analyzed the effect of SBF2-AS1 on cell metastasis. Transwell and matrigel assays showed that the silencing of SBF2-AS1 significantly decreased the metastasis ability of A549 and H1975 cell lines (Fig. 5).

### Silencing of SBF2-AS2 inhibited tumor growth in vivo

**Fig. 6:**
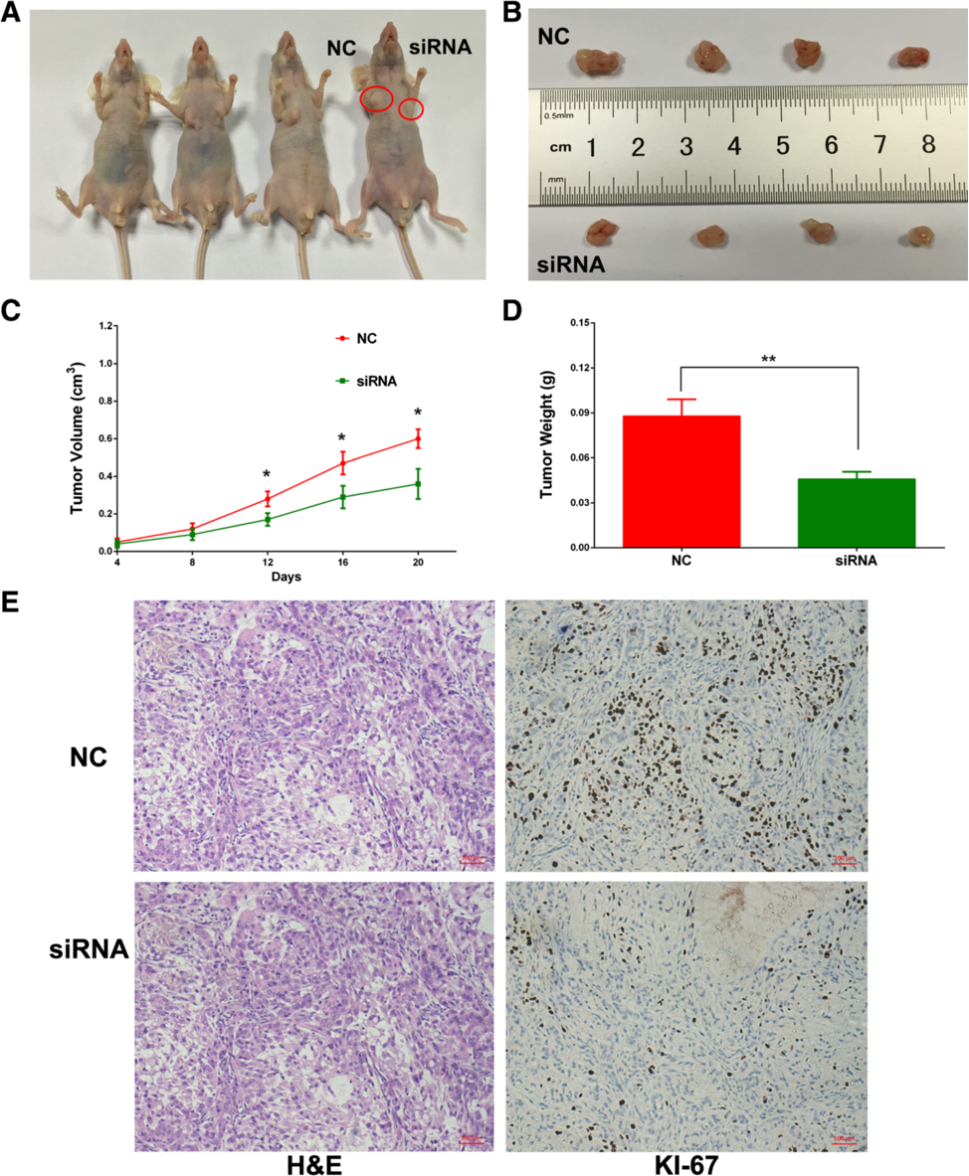
Xenograft tumor models were developed in nude mice by A549 cells transfected with NC and siRNA targeting SBF2-AS1. The xenograft tumor volume (**a, b,** **c**) and weight (**d**) in the SBF2-AS1 siRNA group were significantly lower than those in the NC group. Immunohistochemistry assay showed that Ki-67 staining was weaker in the siRNA group (**e**)

To probe whether SBF2-AS1 regulates NSCLC cell proliferation in vivo, we established xenograft tumor models in nude mice using A549 cells transfected with negative control or siRNA targeting SBF2-AS1. As shown in Fig. 6, SBF2-AS1 silencing inhibited tumor growth in vivo. Xenograft tumors derived from A549 cells transfected with siRNA targeting SBF2-AS1 showed a smaller volume and lower weight than those derived from cells transfected with NC (Fig. 6c, d). In consistence with in vitro results, silence of SBF2-AS1 inhibited proliferation of NSCLC cells, as the proliferation marker Ki-67 was weaker in the siRNA group than the NC group (Fig. 6e).

### SBF2-AS2 inhibited P21 by binding to SUZ12

**Fig. 7:**
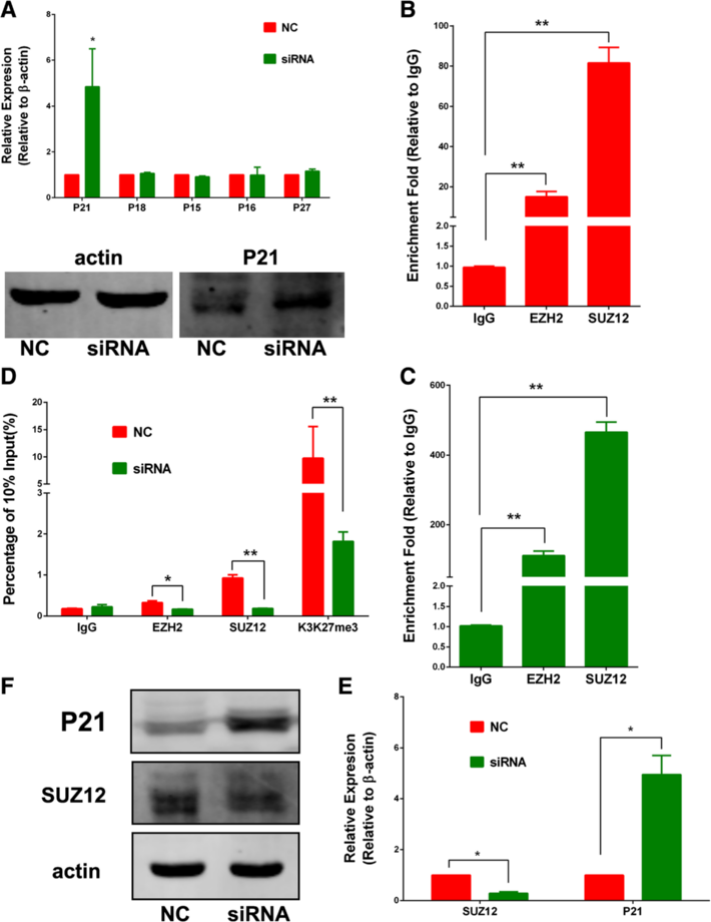
After the silencing of SBF2-AS1, P21 was significantly upregulated as detected by RT-PCR and Western blotting (**a**). The RIP assay demonstrated that SBF2-AS1 could significantly bind to SUZ12 and EZH2 compared with the negative control IgG in A549 (**b**) and H1975 (**c**) cells. ChIP assay showed that the enrichment of SUZ12, EZH2, and H3K27me3 was decreased at the promoter region of P21 by the silencing of SBF2-AS1 in A549 cells (**d**). By silencing SUZ12 in A549 cells, the RNA (**e**) and protein (**f**) levels of P21 were significantly upregulated

Because remarkable G1 cell cycle arrest was observed after the silencing of SBF2-AS1, we analyzed the expression of cyclin-dependent kinase inhibitor (CKI) family genes, key regulators of cell cycle, by qRT-PCR. As shown, we found that P21 was significantly upregulated after the silencing of SBF2-AS1, a finding that was also confirmed at the protein level (Fig. 7a). Thus, it is highly possible that SBF2-AS1 controls the cell cycle by regulating P21.

**Fig. 8:**
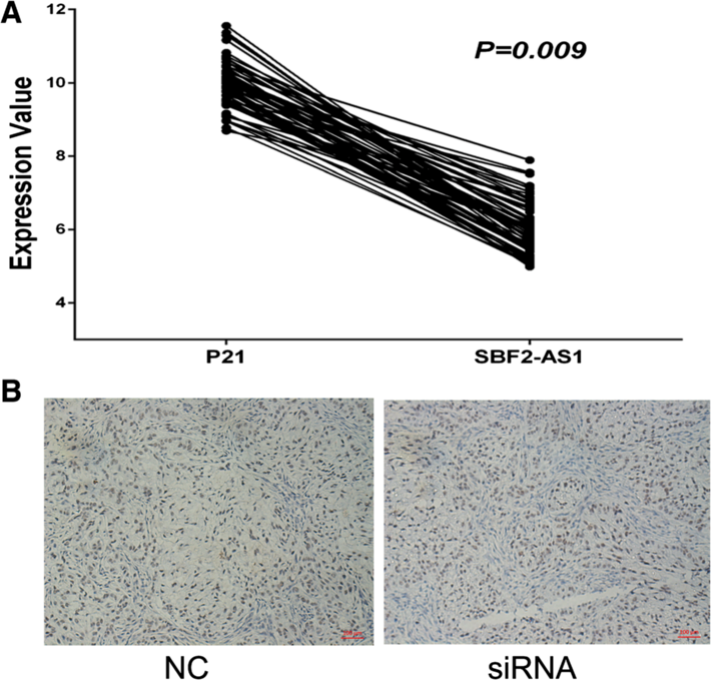
The negative correlation between SBF2-AS1 and P21 was confirmed in the GSE19804 dataset (**a**). In the xenograft tumor tissues derived from siRNA group, P21 staining was stronger than the NC group (**b**)

Currently, most reported lncRNAs have been shown to play roles in transcription regulation, primarily epigenetic modification. Khalil et al. [16] reported that 20 % lncRNA could bind to polycomb repressive complex 2 (PRC2). PRC2 is a critical regulator of histone modification, which negatively regulates gene expression by catalyzing the trimethylation of H3K27. Evidence has proved that that PRC2 is an important driver of tumor development and progression by suppressing various key genes. Thus, we hypothesized that SBF2-AS1 might modulate P21 expression by binding to PRC2. Using an RNA immunoprecipitation (RIP) assay, we confirmed that SBF2-AS1 could bind to SUZ12 and EZH2, the core components of PRC2 (Fig. 7b, c). Compared with the negative control IgG, SBF2-AS1 was significantly enriched by SUZ12 and EZH2 antibodies, and the effect was more evident for SUZ12. In addition to the RIP assay, we performed a chromatin immunoprecipitation (ChIP) assay to investigate whether the silencing of SBF2-AS1 affects the enrichment of PRC2 and trimethylation of histone 3 lysine 27 (H3K27 me3) at the promoter region of P21. As revealed by ChIP assay, after the silencing of SBF2-AS1, the enrichment of SUZ12 and EZH2 was significantly decreased, as well as that of H3K27 me3, at the promoter region of P21 (Fig. 7d). To confirm this finding, we silenced SUZ12 by siRNA, and increased RNA and protein expression of P21 were also observed (Fig. 7e, f). To validate the reverse relationship between SBF2-AS1 and P21, we retrieved the expression values of SBF2-AS1 and P21 from the GSE19804 dataset and found that the expression of the two genes were significantly negatively correlated (*P* = 0.009, Fig. 8a). In addition, in xenograft tumor models immunohistochemistry assay showed that P21 staining was also stronger in the siRNA group (Fig. 8b). Thus, we confirmed that SBF2-AS1 regulated the transcription of P21 by binding to PRC2.

## Discussion

In the current study, we characterized the expression profile of SBF2-AS1 in NSCLC and found that high expression of SBF2-AS1 was associated with advanced TNM stage. By the silencing of SBF2-AS1, the cell proliferation and metastasis ability was inhibited in vitro. The silencing of SBF2-AS1 also inhibited xenograft tumor growth in vivo. Thus, SBF2-AS1 could potentially function as an oncogenic gene in NSCLC.

The mortality rate of lung cancer is higher in males than in females [17]. However, the precise molecular mechanism involved in lung carcinogenesis remains elusive. It has been widely accepted that many lncRNAs transcribed from the human genome have play broad roles in lung cancer [18]. Numerous studies have suggested that lncRNAs are involved in various malignant tumors, such as those of the brain [19, 20], lung [11, 21], breast [22], pancreas [23, 24] and liver [25]. This extends our knowledge concerning carcinogenesis and provides a new pathway in cancer research. For lung cancer, various lung cancer-specific lncRNAs have been identified, such as MALAT1 [26, 27], TARID [28], and LUADT1 [29].

SBF2-AS1 is a novel lncRNA transcribed from chromosome 11p15.4, a finding that has not been reported previously. Firstly, we explored the expression of SBF2-AS1 in NSCLC tissues and analyzed the relationship between the SBF2-AS1 expression level and clinical characteristics, such as gender, tumor size, and TNM stage. As indicated by the microarray data, SBF2-AS1 was upregulated in NSCLC tissues compared with adjacent non-tumor tissues, and the expression was very homogeneous. Similarly, SBF2-AS1 was overexpressed in the 4 NSCLC cell lines analyzed. Statistical analyses revealed that the overexpression of SBF2-AS1 was associated with lymph node metastasis and advanced TNM stage, indicating that SBF2-AS1 could be a biomarker for NSCLC and might be a prognostic factor for survival. The predictive value of SBF2-AS1 should be validated by more experimental evidence.

After siRNA-mediated silencing of SBF2-AS1, we found that SBF2-AS1 could modulate cell proliferation and overexpression of SBF2-AS1 increased proliferation ability of NSCLC cells. In recent years, some lncRNAs, including growth-arrest-specific transcript 5 [30] (GAS5), prostate-specific gene 1 (PCGEM1) [31], prostate-cancer-associated transcript 1 (PCAT-1) [32], and colon cancer-associated transcript 2 (CCAT2) [33], have been reported to regulate tumor cell growth and progression by altering the balance between cell proliferation and apoptosis. In the present study, we also found that SBF2-AS1 influenced tumor cell proliferation by affecting cell cycle distribution. The flow cytometric analysis suggested that the cell cycle was arrested at the G1 phase after transfection with siRNA. According to Khalil et al., most lncRNAs could bind to the PRC2 complex and negatively regulate gene expression at the transcription level [16], such as the lncRNAs HOTAIR [34, 35] and TUG1 [36]. We found that SBF2-AS1 could also bind to the PRC2 complex, particularly the subunit SUZ12. ChIP assay revealed that the silencing of SBF2-AS1 decreased the enrichment of SUZ12 and H3K27 me3 at the promoter region of P21. Thus, these experiments demonstrated that SBF2-AS1 could modulate the cell cycle through epigenetic inhibition of P21.

Metastasis is another important malignant behavior of cancer and is the most troublesome problem in tumor prognosis and therapy. lncRNAs involved in the regulation of tumor metastasis have also been reported, and include MALAT-1 [26] and HOX antisense intergenic RNA (HOTAIR) [35]. Our study suggested that the migration and invasion ability of NSCLC cells were significantly decreased by the silencing of SBF2-AS1, suggesting SBF2-AS1 is also an important regulator of metastasis.

## Conclusion

In this study, we identified a novel lncRNA SBF2-AS1 in NSCLC that is upregulated and correlated with advanced TNM stage. SBF2-AS1 could promote proliferation of NSCLC cells in vitro and in vivo, suggesting that SBF2-AS1 might be an oncogenic lncRNA in NSCLC.

### Ethics approval

This study was approved by the Ethics Boards of the Cancer Institute of Jiangsu Province. Informed written consents were obtained from all patients included in this research.

## Abbreviations

CCK8: Cell Counting Kit-8
ChIP: chromatin immunoprecipitation
lncRNA: long non-coding RNA
ncRNA: non-protein-coding RNA
NSCLC: non-small cell lung cancer
PRC2: polycomb repressive complex 2
RIP: RNA immunoprecipitation
SBF2-AS1: SBF2 antisense RNA 1
